# Nutritional Risk Scores and Cognitive Impairment After Hip Fracture: Strong Associations with Mortality but Limited Discriminative Performance

**DOI:** 10.3390/nu18111741

**Published:** 2026-05-29

**Authors:** Sara Silvaieh, Arastoo Nia, Stephan Heisinger, Domenik Popp

**Affiliations:** 1 Department of Neurology, Medical University of Vienna, Waehringer Guertel 18-20, 1090 Vienna, Austria; 2 Comprehensive Center for Clinical Neurosciences and Mental Health, Medical University of Vienna, Waehringer Guertel 18-20, 1090 Vienna, Austria; 3 Clinical Division of Traumatology, Department of Orthopedics and Trauma Surgery, Medical University of Vienna, Waehringer Guertel 18-20, 1090 Vienna, Austria; arastoo.nia@meduniwien.ac.at (A.N.); stephan.heisinger@meduniwien.ac.at (S.H.); domenik.popp@meduniwien.ac.at (D.P.)

**Keywords:** hip fracture, proximal femur fracture, malnutrition, nutritional screening, mortality, Mini Nutritional Assessment, frailty, cognitive impairment, risk prediction, geriatrics

## Abstract

Background: Malnutrition and cognitive impairment are common in patients with proximal femur fractures and are associated with adverse outcomes. However, the prognostic performance of nutritional screening tools in this population remains uncertain. Methods: In this retrospective cohort study, 1595 patients aged ≥ 60 years undergoing surgery for proximal femur fracture were included. Nutritional status was assessed using seven established scores (MNA, PNI, GNRI, MUST, GMS, NRS-2002, CONUT). Mortality was evaluated at 30 days, 3 months, 6 months, 1 year, and 2 years. Associations were analysed using multivariable logistic regression adjusted for age, sex, ASA class, institutional residence, fracture type, and cognitive impairment. Discrimination was assessed using the area under the curve (AUC), and calibration was evaluated using calibration slopes and Brier scores. Results: Worsening nutritional status was consistently associated with increased mortality across all scores and timepoints. The strongest gradient was observed for MNA, with 2-year mortality increasing from 15.6% in patients with normal nutritional status to 53.8% in malnourished patients. Most scores remained independently associated with mortality after adjustment, with odds ratios per 1 SD deterioration ranging from 1.3 to 1.6. Discriminative performance was modest (AUC 0.57–0.69), with MNA showing the highest values. Differences between scores were small, with overlapping confidence intervals. Calibration was good across all models at 1 and 2 years. Conclusions: Nutritional status is independently associated with mortality after proximal femur fracture but provides limited discrimination for individual risk prediction. Nutritional scores may support identification of vulnerable patients but demonstrated limited performance for individual mortality prediction.

## 1. Introduction

Proximal femur fractures represent a major cause of morbidity and mortality in older adults and constitute an increasing challenge for healthcare systems worldwide [1]. With rising life expectancy and an ageing population, the incidence of hip fractures continues to grow, predominantly affecting elderly individuals following low-energy trauma such as falls from standing height [2]. Approximately 70% of affected patients are women, with a mean age of around 80 years [3]. Despite advances in surgical techniques and perioperative care, outcomes remain poor, with one-year mortality rates ranging from 26% to 36% and substantial long-term loss of independence [4,5].

Outcomes after hip fracture are not determined by the injury alone but reflect a complex interplay of frailty, comorbidity burden, functional reserve, and cognitive status [6]. Cognitive impairment is highly prevalent in this population and has consistently been associated with worse outcomes, including increased mortality, reduced mobility, and higher rates of institutionalisation [7]. At the same time, malnutrition represents another key but often under-recognised determinant of outcome. Nutritional impairment is common in geriatric patients and is associated with delayed recovery, prolonged hospitalisation, and increased mortality [8]. In the context of acute injury and surgical stress, pre-existing malnutrition may further exacerbate catabolic responses and functional decline [9].

Given the often subtle clinical presentation of malnutrition, structured nutritional screening has been advocated as a practical approach to identify high-risk patients. A variety of screening tools have been developed, incorporating anthropometric measures, laboratory parameters, and clinical assessments [10]. However, despite widespread use, no single nutritional score has emerged as a gold standard, and their ability to predict clinically relevant outcomes remains uncertain [11,12]. While numerous studies have demonstrated associations between nutritional status and postoperative mortality, evidence regarding their predictive performance—particularly in comparison across multiple instruments—is limited.

Importantly, nutritional impairment and cognitive dysfunction frequently coexist and may represent complementary dimensions of frailty [13]. Both are associated with reduced physiological reserve, impaired rehabilitation, and increased vulnerability to complications [14]. However, the combined impact of these factors on mortality after proximal femur fracture has not been fully elucidated. Furthermore, it remains unclear whether nutritional screening tools provide meaningful prognostic information beyond established clinical risk factors and whether they are suitable for individual risk prediction.

The aim of this study was therefore to evaluate the association between nutritional status and mortality following surgically treated proximal femur fracture across short- and long-term follow-up. In addition, the discriminative performance and calibration of commonly used nutritional screening tools were assessed to determine their suitability for mortality prediction in this population. Particular attention was given to the relationship between nutritional status and cognitive impairment, with the objective of clarifying their combined role in defining patient vulnerability.

## 2. Methods

### 2.1. Study Design and Population

This retrospective observational cohort study was conducted at the Clinical Division of Traumatology, Department of Orthopaedics and Trauma Surgery, Medical University of Vienna, Vienna, Austria, a level 1 trauma centre. All patients aged 60 years or older who were admitted with a proximal femur fracture and underwent operative treatment between January 2017 and January 2020 were screened for inclusion.

Eligible fractures comprised femoral neck, pertrochanteric, and subtrochanteric fractures. Patients treated non-operatively, those with periprosthetic or pathological fractures related to active neoplastic disease, polytrauma, or patients with missing baseline information precluding classification of cognitive status or calculation of nutritional scores were excluded from analysis. Pathological fractures related to malignant disease were excluded from analysis. Patients with surgical delay exceeding 48 h were excluded in order to minimise confounding related to delayed operative treatment and associated mortality risk.

The study was conducted in accordance with the STROBE recommendations for observational studies. Ethical approval was obtained from the institutional review board (EK 1655/2020), and the requirement for informed consent was waived owing to the retrospective design.

### 2.2. Data Sources and Baseline Variables

Data were extracted from the institutional electronic medical record system and analysed in pseudonymised form. Baseline variables included age, sex, body mass index (BMI), American Society of Anesthesiologists (ASA) class, institutional residence, fracture type, surgical treatment, and comorbidities.

Fractures were categorised as femoral neck (medial or lateral), pertrochanteric, or subtrochanteric. Surgical treatment was classified as intramedullary fixation, hemiarthroplasty, total hip arthroplasty, dynamic hip screw, or cannulated screw fixation. Comorbidities included broad clinically relevant categories such as cardiac disease, chronic obstructive pulmonary disease, renal disease, and diabetes mellitus to characterise the overall medical burden and frailty profile of the cohort.

Renal disease was defined according to documented pre-existing renal comorbidity within the medical record. A standardised eGFR-based stratification was consistently available retrospectively as it is standard procedure for perioperative blood work.

### 2.3. Cognitive Impairment

Baseline cognitive status was grouped into three categories: no impairment, mild cognitive impairment, and dementia. Classification was based on a review of the medical records at admission, taking into account previously documented diagnoses (e.g., dementia or mild cognitive impairment), results from cognitive screening tools such as the Mini-Mental State Examination (MMSE), when available, and contemporaneous preoperative clinical documentation of cognitive function at presentation.

If a diagnosis of dementia or MCI had already been established, this classification was used. In patients without a formal diagnosis, MMSE scores were used to estimate cognitive status. Although an MMSE score below 24 is commonly considered indicative of cognitive impairment, a more detailed categorization was applied for analysis: scores ≥ 27 were considered normal, 24–26 as mild impairment, and ≤23 as dementia. This categorization was used for analytical purposes and does not represent a formal diagnosis. As MMSE data were not available in all patients, classification was based on the integration of all available routinely documented preoperative clinical information, including previously established diagnoses, cognitive screening results where available, and contemporaneous admission documentation. Only preoperative cognitive assessments and admission documentation available at presentation were considered for classification of cognitive status.

Patients without evidence of cognitive impairment served as the reference group. This approach was intended to reflect routinely available clinical information within a retrospective geriatric trauma cohort and to provide standardised epidemiological stratification across the study population.

### 2.4. Nutritional Assessment

Nutritional status at admission was assessed using seven established screening and risk scores: the Mini Nutritional Assessment Short Form (MNA-SF), Prognostic Nutritional Index (PNI), Geriatric Nutritional Risk Index (GNRI), Malnutrition Universal Screening Tool (MUST), Graz Malnutrition Screening (GMS), Nutritional Risk Screening (NRS-2002), and Controlling Nutritional Status (CONUT) score.

The scores were calculated using the earliest available clinical and laboratory data obtained at admission. Established cut-offs were applied to categorise patients into nutritional risk groups. The MNA-SF was categorised according to established cut-offs into normal nutritional status (12–14), at risk of malnutrition (8–11), and malnourished (0–7). For GNRI, the patients were classified as no risk (≥98), low risk (92–<98), moderate risk (82–<92), and severe risk (<82). For PNI, values > 38 were considered normal, 35–38 moderate malnutrition, and <35 severe malnutrition. MUST was categorised as 0 (no risk), 1 (moderate risk), and ≥2 (high risk). GMS was categorised as no risk (0–2) and at risk (>2). NRS-2002 was dichotomised as <3 (no risk) and ≥3 (risk). CONUT was categorised as normal (0–1), mild (2–4), moderate (5–8), and severe (9–12).

### 2.5. Surgical Management

The patients were managed according to an institutional hip fracture pathway. Treatment decisions were based on fracture type, biological age, pre-fracture mobility, and overall medical condition. Femoral neck fractures were treated with internal fixation or arthroplasty, whereas extracapsular fractures were managed predominantly with intramedullary fixation or, in selected cases, dynamic hip screw constructs. Perioperative management followed a multidisciplinary approach involving trauma surgeons, anaesthesiologists, and internal medicine specialists.

### 2.6. Outcomes

The primary outcome was all-cause mortality within 2 years following surgery. Mortality was additionally assessed at predefined timepoints of 30 days, 3 months, 6 months, and 1 year. Time-to-event was defined as the interval between the date of surgery and the date of death or censoring at the last available follow-up.

### 2.7. Statistical Analysis

Continuous variables are presented as mean (SD) or median (range), as appropriate. Categorical variables are reported as *n* (%). Mortality proportions were calculated for each timepoint.

Associations between nutritional status and mortality were evaluated using univariable and multivariable logistic regression models at each predefined timepoint. Odds ratios (ORs) with 95% confidence intervals were reported per 1 SD worsening of nutritional status. Multivariable models were adjusted for age, sex, ASA class, institutional residence, fracture type, and cognitive impairment.

Discriminatory performance of each nutritional score was assessed using receiver operating characteristic (ROC) analysis, with calculation of the area under the curve (AUC) and corresponding 95% confidence intervals at each timepoint. Pairwise comparisons of AUCs were performed using DeLong’s test with Holm correction for multiple testing.

Calibration was assessed at 1 and 2 years using calibration slopes and Brier scores. A calibration slope of 1.0 indicates perfect agreement between predicted and observed risk, whereas lower Brier scores indicate better overall prediction accuracy.

All the statistical tests were two-sided, and a *p* value < 0.05 was considered statistically significant. The analyses were performed using SPSS software (version 21, IBM Corp., Armonk, NY, USA).

## 3. Results

Of 1595 patients, 486 (30.5%) had any form of cognitive impairment at baseline, including 324 (20.3%) with mild cognitive impairment and 162 (10.2%) with dementia. Patients with cognitive impairment were older (83.4 years [SD 9.5] in cognitive impairment vs. 79.9 years [SD 9.1] without impairment) and had a higher burden of systemic disease. The distribution of ASA class shifted towards more severe disease, with a greater proportion of ASA III–IV in the impaired groups. Institutionalisation was more frequent among cognitively impaired patients, affecting 79 (48.8%) of those with dementia compared with 155 (14.0%) without impairment (Table 1). Fracture patterns differed modestly, with pertrochanteric fractures more common in patients with cognitive impairment, accompanied by more frequent use of intramedullary fixation. Comorbidities, including chronic obstructive pulmonary disease, renal disease, and diabetes mellitus, were more prevalent in cognitively impaired patients. Body mass index decreased slightly with increasing cognitive impairment.

Nutritional status differed according to cognitive impairment (Table 2). The most pronounced gradient was observed for MNA, decreasing from a median of 12 in patients without cognitive impairment to 9 in mild cognitive impairment and 8 in dementia. Similar patterns were observed for CONUT, GMS, and NRS, whereas differences for PNI and GNRI were smaller.

Mortality increased stepwise with worsening nutritional status across all scores and timepoints (Table 3). The strongest gradient was observed for MNA, with 30-day mortality increasing from 3.0% in patients with normal nutritional status to 11.9% in malnourished patients, and 2-year mortality from 15.6% to 53.8%. Similar gradients were observed for PNI, GNRI, and CONUT. For MUST and NRS, patients classified at risk showed consistently higher mortality at all timepoints compared with those without risk.

In multivariable analyses, worsening nutritional status remained independently associated with mortality after adjustment for age, sex, ASA class, institutionalisation, fracture type, and cognitive impairment (Table 4). For MNA, each 1 SD deterioration was associated with increased mortality at 30 days (OR 1.46, 95% CI 1.12–1.91) and 2 years (OR 1.48, 1.28–1.71). Comparable associations were observed for PNI, GMS, NRS, and CONUT, with effect sizes generally ranging from OR 1.3 to 1.6. GNRI showed smaller effect sizes but remained significantly associated with mortality.

Discriminative performance of all nutritional scores was modest (Table 5). AUC values ranged from 0.57 to 0.69 across timepoints. MNA demonstrated the highest discrimination from 3 months onward (AUC 0.68–0.69), whereas GNRI, MUST, and NRS showed lower performance. Differences between scores were small, with largely overlapping confidence intervals.

Pairwise comparisons using DeLong testing with Holm correction showed modest differences between scores. MNA demonstrated higher discrimination compared with MUST across timepoints and compared with NRS and CONUT at later follow-up, whereas differences between MNA, PNI, and GMS were not statistically significant after correction.

Calibration was good for all scores at 1 and 2 years (Table 6). Calibration slopes were close to 1.0 across models, and Brier scores were similar between scores (0.156–0.162 at 1 year and 0.198–0.210 at 2 years), indicating comparable overall prediction error.

## 4. Discussion

In this cohort of patients undergoing surgery for proximal femur fracture, nutritional status was consistently associated with both short- and long-term mortality. Across all evaluated screening tools, worsening nutritional status was accompanied by a stepwise increase in mortality risk, which remained significant after adjustment for established clinical factors. However, despite these robust associations, the ability of nutritional scores to discriminate individual patient risk was limited, with AUC values remaining below thresholds typically considered clinically useful.

A central finding of this study is the clear gradient between nutritional impairment and mortality. This was most pronounced for MNA, where mortality increased more than threefold between patients with normal nutritional status and those classified as malnourished at 2 years. Comparable, although less pronounced, gradients were observed for PNI, GNRI, and CONUT. These findings are consistent with prior studies demonstrating an association between malnutrition and adverse outcomes following hip fracture [15,16,17], and extend this evidence by providing a detailed time-dependent analysis across multiple validated nutritional instruments. The observation that risk separation was already evident at 30 days and widened over time suggests that nutritional status may reflect early postoperative vulnerability and longer-term recovery potential.

The persistence of these associations after multivariable adjustment indicates that nutritional status captures clinically relevant information beyond traditional risk factors such as age, comorbidity burden, and fracture characteristics. Similar observations have been reported in previous studies evaluating nutritional indices in surgical and geriatric populations [18,19]. However, the present findings also highlight an important limitation: while nutritional scores are sensitive markers of vulnerability, their specificity for mortality prediction remains limited. This likely reflects the multifactorial nature of mortality in this population, where nutritional status represents only one component within a broader syndrome of frailty.

Notably, despite consistent associations across scores, their discriminative performance was modest. MNA showed the highest AUC values, particularly at intermediate and longer-term follow-up, but differences between scores were small and confidence intervals largely overlapping. These findings are in line with previous work reporting only moderate predictive performance of nutritional screening tools in older populations [19,20]. The discrepancy between strong statistical association and limited discrimination underscores the distinction between identifying risk factors and accurately predicting individual outcomes. In this context, nutritional scores appear better suited for risk stratification at the population level rather than for precise individual prediction.

Calibration analysis provides additional insight into the performance of these models. All scores demonstrated good agreement between predicted and observed mortality at 1 and 2 years, with calibration slopes close to 1.0 and similar Brier scores across models. This finding indicates that, despite limited discrimination, nutritional scores provide reliable estimates of absolute risk at the group level. Similar discrepancies between discrimination and calibration have been observed in other clinical prediction models [10,21], emphasising that reliance on AUC alone may underestimate the clinical utility of a model. In practice, well-calibrated models may still provide supportive group-level prognostic information when interpreted alongside broader clinical assessment.

The comparatively stronger performance of MNA may be explained by its design.

The stronger performance of MNA may partly reflect its multidimensional structure. Unlike laboratory-based indices, MNA includes functional and neuropsychological items, which may overlap with frailty and cognitive impairment. Therefore, its association with mortality should not be interpreted as reflecting nutritional status alone.

Unlike purely laboratory-based scores such as PNI and CONUT, MNA incorporates functional and cognitive components, including mobility and neuropsychological status [22]. Given that cognitive impairment is itself a major determinant of outcome after hip fracture, the inclusion of these domains likely enhances the ability of MNA to capture global vulnerability [23,24]. This is particularly relevant in the present cohort, where a substantial proportion of the patients exhibited cognitive impairment, and where nutritional and cognitive deficits frequently coexisted [25]. This overlap may partly explain the strong associations with mortality despite only modest discriminative performance for individual risk prediction.

The interaction between cognitive impairment and nutritional status warrants particular consideration. Both conditions reflect underlying frailty and are associated with reduced physiological reserve, impaired recovery, and increased susceptibility to complications. Nutritional impairment in this population likely reflects a broader syndrome of frailty, sarcopenia, functional dependency, and cognitive decline rather than isolated nutritional deficiency alone. Previous work has demonstrated that cognitive impairment independently predicts mortality after hip fracture [22,23,24], and the present findings suggest that nutritional impairment may represent a complementary dimension of risk.

MCI and dementia represent clinically distinct stages, and their prognostic implications may differ. In the present study, both were associated with poorer nutritional status, but the strongest vulnerability profile was observed in the patients with documented dementia.

Patients with cognitive impairment, particularly those with dementia, are at increased risk of malnutrition due to reduced appetite and oral intake, dysphagia, impaired meal initiation, and dependency in feeding, as well as neuropsychiatric symptoms and limited participation in rehabilitation [11,13]. These mechanisms may partly explain the overlap between cognitive impairment, nutrition and mortality after hip fracture.

From a clinical perspective, these findings support the routine assessment of nutritional status in patients with proximal femur fractures. While nutritional scores alone may not be sufficient for precise mortality prediction, they provide valuable information for identifying high-risk patients who may benefit from targeted interventions. Previous studies have shown that structured nutritional management and interdisciplinary care can improve outcomes, including reductions in complications and length of hospital stay [26]. Early identification of patients at risk may therefore facilitate the timely implementation of such strategies.

## 5. Limitations

Several limitations should be considered. The retrospective single-centre design introduces the possibility of residual confounding and may limit generalizability to other healthcare systems and populations. Nutritional impairment likely overlaps substantially with frailty, sarcopenia, functional dependency, and cognitive decline, which could not be fully disentangled within the present study design. Because cognitive assessment was based on retrospective routine clinical documentation, some degree of heterogeneity in classification cannot be fully excluded. Furthermore, although patients with missing baseline information required for nutritional score calculation or cognitive classification were excluded, retrospective routine clinical documentation may still introduce variability in data completeness. Finally, detailed characterisation of dementia subtype and severity was not consistently available across the cohort.

## 6. Conclusions

Nutritional status is strongly associated with short- and long-term mortality following surgically treated proximal femur fracture, independent of established clinical risk factors, including cognitive impairment. All evaluated nutritional scores demonstrated consistent risk gradients, with MNA showing the most pronounced associations. However, despite these robust relationships, discriminative performance remained modest, and no score achieved sufficient accuracy for stand-alone mortality prediction. In contrast, calibration was good across models, indicating reliable estimation of group-level risk.

These findings suggest that nutritional scores should be interpreted as markers of overall vulnerability rather than as isolated indicators of nutritional status. This is particularly relevant in patients with cognitive impairment, specifically dementia, in whom nutritional risk likely reflects broader frailty and functional decline. Routine nutritional assessment may support the identification of vulnerable patients and complement broader multidimensional clinical assessment, particularly in the context of coexisting cognitive impairment. Future studies should focus on integrating nutritional assessment with cognitive and functional measures and evaluating the impact of targeted nutritional interventions in this highly vulnerable population.

## Figures and Tables

**Table 1:** Baseline characteristics.

Characteristics	Total (*n* = 1595)	No Impairment (*n* = 1109)	MCI (*n* = 324)	Dementia (*n* = 162)
**Age (years)**	81.4 ± 9.3	79.9 ± 9.1	82.8 ± 9.2	83.4 ± 9.5
Median (range)	82 (60–106)	80 (60–104)	83 (61–105)	84 (62–106)
**Female sex**	1137 (70.3%)	795 (71.6%)	229 (70.6%)	113 (69.8%)
**BMI (kg/m^2^)**	23.1 ± 4.2	23.4 ± 4.1	22.8 ± 4.3	22.6 ± 4.5
Median (range)	22.8 (14–38)	23.1 (15–37)	22.5 (14–38)	22.3 (14–36)
**ASA classification**				
ASA I	53 (3.3%)	40 (3.6%)	11 (3.4%)	2 (1.2%)
ASA II	586 (36.6%)	478(43.1%)	86 (26.5%)	22 (13.6%)
ASA III	854 (53.6%)	540 (48.6%)	199 (61.5%)	115 (70.9%)
ASA IV	100 (6.4%)	50 (4.6%)	27 (8.3%)	23 (14.3%)
ASA V	2 (0.1%)	1 (0.1%)	1 (0.3%)	0 (0.0%)
**Institutionalised**	362 (22.0%)	155 (14%)	128 (39.5%)	79 (48.8)
**Fracture type**				
Femoral neck (medial)	790 (49.5%)	580 (52.3%)	139 (42.9%)	71 (43.8%)
Femoral neck (lateral)	17 (1.1%)	12 (1.1%)	3 (0.9%)	2 (1.2%)
Pertrochanteric	768 (48.3%)	505 (45.6%)	176 (54.3%)	87 (53.8%)
Subtrochanteric	20 (1.1%)	12 (1.1%)	6 (1.9%)	2 (1.2%)
**Surgical treatment**				
IM nail	760 (50.3%)	505 (45.5%)	164 (49.4%)	91 (56.2%)
HEP	512 (31.8%)	360 (32.4%)	99 (34.9%)	53 (32.7%)
THA	91 (5.6%)	71 (6.5%)	16 (1.2%)	4 (2.5%)
DHS	85 (5.2%)	62 (5.6%)	16 (6.8%)	7 (4.3%)
CS	147 (7.1%)	111 (10.0%)	29 (7.7%)	7 (4.3%)
**Comorbidities**				
Cardiac disease	310 (19.4%)	180 (16.2%)	84(25.9%)	46 (28.4%)
COPD	244 (15.3%)	132 (11.9%)	69 (21.3%)	43 (26.5%)
Renal disease	163 (10.2%)	74 (6.7%)	53 (16.3%)	36 (22.2%)
Diabetes mellitus	343 (21.5%)	212 (19.1%)	85 (26.2%)	46 (28.4%)

Data are presented as mean ± standard deviation, median (range), or number (percentage). MCI = mild cognitive impairment; BMI = body mass index; ASA = American Society of Anesthesiologists; COPD = chronic obstructive pulmonary disease; IM = intramedullary nailing; HEP = hemiarthroplasty; THA = total hip arthroplasty; DHS = dynamic hip screw; CS = cannulated screw.

**Table 2:** Nutritional scores according to cognitive impairment.

Score	Total	No Impairment (*n* = 1109)	MCI (*n* = 324)	Dementia (*n* = 162)
MNA	11 (9–12)	12 (10–13)	9 (7–10)	8 (6–9)
PNI	38.3 (34.3–42.3)	38.8 (34.9–43.0)	37.2 (33.4–40.3)	37.1 (34.0–41.8)
GNRI	88.8 (83.1–94.3)	89.3 (83.7–94.9)	87.1 (81.9–92.8)	87.0 (82.5–92.9)
MUST	0 (0–1)	0 (0–0)	0 (0–1)	0 (0–1)
GMS	2 (2–3)	2 (1–3)	2 (2–4)	2 (2–3)
NRS	2 (2–3)	2 (2–3)	2 (2–4)	2 (2–3)
CONUT	5 (3–7)	5 (3–7)	6 (4–7)	6 (4–7)

MNA = Mini Nutritional Assessment; PNI = Prognostic Nutritional Index; GNRI = Geriatric Nutritional Risk Index; MUST = Malnutrition Universal Screening Tool; GMS = Graz Malnutrition Screening; NRS = Nutritional Risk Screening; CONUT = Controlling Nutritional Status.

**Table 3:** Mortality according to nutritional risk.

Score	Category	*n*	30 Days	3 Months	6 Months	1 Year	2 Years
MNA	Normal	596	18 (3.0%)	29 (4.9%)	38 (6.4%)	58 (9.7%)	93 (15.6%)
MNA	At risk	748	37 (4.9%)	85 (11.4%)	121 (16.2%)	172 (23.0%)	266 (35.6%)
MNA	Malnourished	253	30 (11.9%)	62 (24.5%)	82 (32.4%)	107 (42.3%)	136 (53.8%)
PNI	Normal (>38)	821	27 (3.3%)	49 (6.0%)	74 (9.0%)	114 (13.9%)	176 (21.4%)
PNI	Moderate (35–38)	315	15 (4.8%)	40 (12.7%)	54 (17.1%)	78 (24.8%)	115 (36.5%)
PNI	Severe (<35)	452	43 (9.5%)	87 (19.2%)	113 (25.0%)	145 (32.1%)	204 (45.1%)
GNRI	No risk (≥98)	236	8 (3.4%)	15 (6.4%)	21 (8.9%)	32 (13.6%)	54 (22.9%)
GNRI	Low risk (92–<98)	325	15 (4.6%)	22 (6.8%)	33 (10.2%)	49 (15.1%)	77 (23.7%)
GNRI	Moderate risk (82–<92)	697	30 (4.3%)	73 (10.5%)	99 (14.2%)	140 (20.1%)	208 (29.8%)
GNRI	Severe risk (<82)	333	32 (9.6%)	66 (19.8%)	88 (26.4%)	116 (34.8%)	156 (46.8%)
MUST	No risk (0)	1192	44 (3.7%)	101 (8.5%)	139 (11.7%)	205 (17.2%)	324 (27.2%)
MUST	Moderate risk (1)	185	19 (10.3%)	31 (16.8%)	41 (22.2%)	58 (31.4%)	77 (41.6%)
MUST	High risk (≥2)	219	21 (9.6%)	43 (19.6%)	60 (27.4%)	73 (33.3%)	93 (42.5%)
GMS	No risk (0–2)	1035	39 (3.8%)	83 (8.0%)	112 (10.8%)	162 (15.7%)	256 (24.7%)
GMS	At risk (3)	281	16 (5.7%)	35 (12.5%)	49 (17.4%)	73 (26.0%)	107 (38.1%)
GMS	Malnourished (>3)	279	29 (10.4%)	57 (20.4%)	79 (28.3%)	101 (36.2%)	131 (47.0%)
NRS	No risk (<3)	1086	40 (3.7%)	86 (7.9%)	121 (11.1%)	178 (16.4%)	278 (25.6%)
NRS	Risk (≥3)	508	44 (8.7%)	89 (17.5%)	119 (23.4%)	158 (31.1%)	216 (42.5%)
CONUT	Normal (0–1)	94	1 (1.1%)	4 (4.3%)	7 (7.4%)	11 (11.7%)	17 (18.1%)
CONUT	Mild (2–4)	573	21 (3.7%)	37 (6.5%)	55 (9.6%)	89 (15.5%)	144 (25.1%)
CONUT	Moderate (5–8)	746	38 (5.1%)	94 (12.6%)	128 (17.2%)	177 (23.7%)	252 (33.8%)
CONUT	Severe (9–12)	184	25 (13.6%)	41 (22.3%)	51 (27.7%)	60 (32.6%)	82 (44.6%)

MNA = Mini Nutritional Assessment; PNI = Prognostic Nutritional Index; GNRI = Geriatric Nutritional Risk Index; MUST = Malnutrition Universal Screening Tool; GMS = Graz Malnutrition Screening; NRS = Nutritional Risk Screening; CONUT = Controlling Nutritional Status.

**Table 4:** Logistic regression analysis of mortality at predefined timepoints.

Score	Model	30 Days	3 Months	6 Months	1 Year	2 Years
**MNA**	Univariable	1.63 (1.34–1.99)	1.81 (1.56–2.10)	1.87 (1.64–2.14)	1.88 (1.66–2.12)	1.83 (1.64–2.05)
	Multivariable	1.46 (1.12–1.91)	1.58 (1.30–1.92)	1.71 (1.43–2.03)	1.62 (1.39–1.90)	1.48 (1.28–1.71)
**PNI**	Univariable	1.74 (1.38–2.18)	1.79 (1.52–2.12)	1.75 (1.51–2.04)	1.66 (1.45–1.89)	1.67 (1.49–1.88)
	Multivariable	1.56 (1.22–2.00)	1.63 (1.35–1.96)	1.59 (1.36–1.87)	1.50 (1.30–1.72)	1.51 (1.33–1.71)
**GNRI**	Univariable	1.53 (1.24–1.89)	1.68 (1.43–1.96)	1.66 (1.44–1.92)	1.58 (1.39–1.79)	1.54 (1.37–1.72)
	Multivariable	1.38 (1.10–1.74)	1.56 (1.31–1.85)	1.55 (1.33–1.81)	1.47 (1.28–1.68)	1.42 (1.25–1.61)
**MUST**	Univariable	1.46 (1.24–1.72)	1.43 (1.26–1.62)	1.50 (1.34–1.68)	1.41 (1.27–1.57)	1.32 (1.19–1.46)
	Multivariable	1.41 (1.18–1.69)	1.37 (1.19–1.57)	1.44 (1.28–1.64)	1.35 (1.20–1.52)	1.24 (1.11–1.38)
**GMS**	Univariable	1.67 (1.39–2.01)	1.62 (1.41–1.86)	1.72 (1.52–1.96)	1.68 (1.49–1.89)	1.63 (1.46–1.82)
	Multivariable	1.59 (1.29–1.95)	1.48 (1.27–1.73)	1.60 (1.39–1.84)	1.53 (1.35–1.74)	1.45 (1.28–1.63)
**NRS**	Univariable	1.56 (1.29–1.88)	1.51 (1.31–1.73)	1.57 (1.38–1.77)	1.50 (1.34–1.68)	1.47 (1.32–1.63)
	Multivariable	1.46 (1.18–1.79)	1.39 (1.19–1.63)	1.46 (1.27–1.68)	1.39 (1.23–1.58)	1.33 (1.18–1.50)
**CONUT**	Univariable	1.77 (1.43–2.20)	1.73 (1.48–2.03)	1.60 (1.40–1.84)	1.49 (1.32–1.68)	1.49 (1.34–1.66)
	Multivariable	1.58 (1.26–1.98)	1.56 (1.32–1.84)	1.44 (1.24–1.67)	1.33 (1.17–1.52)	1.33 (1.18–1.50)

MNA = Mini Nutritional Assessment; PNI = Prognostic Nutritional Index; GNRI = Geriatric Nutritional Risk Index; MUST = Malnutrition Universal Screening Tool; GMS = Graz Malnutrition Screening; NRS = Nutritional Risk Screening; CONUT = Controlling Nutritional Status.

**Table 5:** Discriminative performance of nutritional scores for mortality across timepoints.

Score	30 Days	3 Months	6 Months	1 Year	2 Years
MNA	0.64 (0.58–0.70)	0.68 (0.64–0.72)	0.68 (0.65–0.72)	0.69 (0.66–0.72)	0.68 (0.65–0.70)
PNI	0.65 (0.59–0.71)	0.66 (0.62–0.70)	0.66 (0.62–0.70)	0.64 (0.61–0.67)	0.64 (0.61–0.67)
GNRI	0.63 (0.56–0.69)	0.65 (0.61–0.69)	0.65 (0.61–0.68)	0.63 (0.60–0.66)	0.62 (0.59–0.65)
MUST	0.62 (0.57–0.67)	0.60 (0.56–0.64)	0.60 (0.57–0.64)	0.59 (0.56–0.62)	0.57 (0.54–0.59)
GMS	0.65 (0.59–0.71)	0.65 (0.61–0.69)	0.66 (0.62–0.69)	0.65 (0.62–0.68)	0.64 (0.61–0.67)
NRS	0.62 (0.56–0.68)	0.62 (0.58–0.66)	0.63 (0.59–0.66)	0.62 (0.59–0.65)	0.61 (0.58–0.63)
CONUT	0.65 (0.59–0.71)	0.65 (0.61–0.69)	0.63 (0.60–0.68)	0.61 (0.58–0.65)	0.61 (0.58–0.64)

MNA = Mini Nutritional Assessment; PNI = Prognostic Nutritional Index; GNRI = Geriatric Nutritional Risk Index; MUST = Malnutrition Universal Screening Tool; GMS = Graz Malnutrition Screening; NRS = Nutritional Risk Screening; CONUT = Controlling Nutritional Status.

**Table 6:** Calibration of nutritional scores for mortality prediction at 1 and 2 years.

Score	1 Year Slope	1 Year Brier	2 Year Slope	2 Year Brier
MNA	1.00	0.156	0.99	0.198
PNI	0.95	0.161	0.98	0.204
GNRI	0.98	0.161	0.97	0.206
MUST	1.00	0.162	0.96	0.210
GMS	0.99	0.158	1.00	0.203
NRS	0.96	0.161	0.99	0.207
CONUT	0.97	0.162	0.96	0.207

MNA = Mini Nutritional Assessment; PNI = Prognostic Nutritional Index; GNRI = Geriatric Nutritional Risk Index; MUST = Malnutrition Universal Screening Tool; GMS = Graz Malnutrition Screening; NRS = Nutritional Risk Screening; CONUT = Controlling Nutritional Status.

## Data Availability

The data that support the findings of this study are available on request from the corresponding author, D.P. The data are not publicly available due to restrictions containing information that could compromise the privacy of research participants.
